# Modeling of Chronic Myeloid Leukemia: An Overview of* In Vivo* Murine and Human Xenograft Models

**DOI:** 10.1155/2016/1625015

**Published:** 2016-08-25

**Authors:** Pallavi Sontakke, Jenny Jaques, Edo Vellenga, Jan Jacob Schuringa

**Affiliations:** Department of Experimental Hematology, Cancer Research Center Groningen, University Medical Center Groningen, University of Groningen, Groningen, Netherlands

## Abstract

Over the past years, a wide variety of* in vivo* mouse models have been generated in order to unravel the molecular pathology of Chronic Myeloid Leukemia (CML) and to develop and improve therapeutic approaches. These models range from (conditional) transgenic models, knock-in models, and murine bone marrow retroviral transduction models followed by transplantation. With the advancement of immunodeficient xenograft models, it has become possible to use human stem/progenitor cells for* in vivo* studies as well as cells directly derived from CML patients. These models not only mimic CML but also have been instrumental in uncovering various fundamental mechanisms of CML disease progression and tyrosine kinase inhibitor (TKI) resistance. With the availability of iPSC technology, it has become feasible to derive, maintain, and expand CML subclones that are at least genetically identical to those in patients. The following review provides an overview of all murine as well as human xenograft models for CML established till date.

## 1. Introduction

CML is a myeloproliferative disorder characterized by accumulation of several types of myeloid precursor cells. CML is caused by a reciprocal translocation between chromosomes 9 and 22, also known as the Philadelphia (Ph^+^) chromosome, which leads to the formation of BCR-ABL fusion protein depending on precise break point and splicing of final BCR-ABL mRNA [1]. More than 90% of the patients are diagnosed at a relatively early stage of the disease known as chronic phase (CP). It is generally accepted that acquisition of the t(9;22) BCR-ABL translocation is the initiating event in the CML CP [2, 3]. It is believed that this acquisition initially occurs in a single HSC that gains proliferative advantage and/or aberrant differentiation capacity over the normal cells, giving rise to expansion of the myeloid compartment [4, 5]. Before the discovery of the tyrosine kinase inhibitors (TKIs), all patients with CML-CP progressed to advanced disease in a median of 5 years after treatment. This phase is divided into an accelerated phase (AP) followed by a myeloid blast crisis (BC) [6], although transition to a lymphoid blast crisis can occur as well. The molecular mechanisms underlying this disease progression are still not entirely understood, but it is likely that they involve activation of oncogenic factors and inactivation of tumor suppressors. The phenotype of the self-renewing leukemic stem cells that maintain CML remains obscure. In CML-CP, LSCs reside within Lin^−^CD34^+^38^−^ fraction, suggesting that the first cell that gains the BCR-ABL translocation is a stem cell or immature progenitor cell [7–9]. By performing engraftment studies in SCID (severe combined immunodeficiency) mice, Cobaleda et al. showed that the self-renewal and phenotypic properties of leukemia-initiating cells (SL-ICs) in BCR-ABL p190 Ph-ALL were similar to those of normal stem cells, suggesting that HSCs act as cell of origin in CML [10]. Many other studies have also supported this idea and established a basic understanding of the CML hierarchy [11, 12]. Furthermore, in contrast to other oncogenes like MOZ-TIF2 and MLL-ENL, BCR-ABL cannot confer self-renewal properties on committed progenitor cells, again suggesting that immature stem/progenitor cells are most likely the cell of origin in CML [13–15]. Upon progression to BC-AML, it has been shown that the phenotype of the leukemia-maintaining stem cell changes and starts to resemble the phenotype of granulocyte/macrophage progenitors (GMPs). Jamieson et al. reported* in vitro* self-renewal capacity of leukemic GMPs due to increased levels of nuclear *β*-Catenin compared to normal GMPs [16]. Furthermore, Minami et al. provided evidence that BCR-ABL transformed GMPs with abnormal *β*-Catenin activity can function as LSCs that maintain the disease [17]. These data demonstrate that LSCs that initiate and later on maintain CML can possess versatile characteristics that change upon progression of the disease.

Treatment of CML patients with the inhibitor imatinib leads to response rates of over 95% [18]. Yet, the leukemia-initiating cells are not targeted efficiently [12, 19, 20], and patients might need to stay on therapy lifelong. A significant proportion of patients develop primary resistance to therapy, often due to mutations in the activation loop, phosphate binding P-loop, or the catalytic kinase domain of BCR-ABL [21–23]. Second and third generation TKIs have been developed such as dasatinib, nilotinib, ponatinib, and bafetinib but TKI resistance appears to remain a challenge. Furthermore, acquired resistance during the course of therapy due to whether resistant clonal selection by protective microenvironment, increased efflux of drug, or BCR-ABL signaling mechanism renders TKI ineffective for CML treatment [24–26]. Thus, identification of additional targets that facilitate the eradication of BCR-ABL^+^ leukemia-initiating cells is needed.

**Figure 1:**
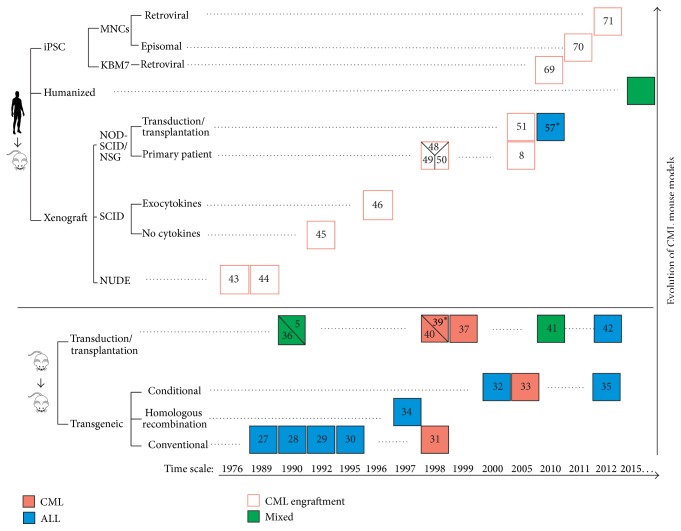
CML human and murine models till date. This figure summarizes established models for CML using chimeric mouse strains as well as different immunocompromised strains. Different experimental approaches and strategies along the time are also highlighted. “*∗*” refers to generation of serially transplantable leukemia and reference numbers (as indicated in Table 1) are denoted for each mouse model.

Having assessed the cause of disease, the goal lies in identifying characteristics of remnant CML LSCs which lead to disease progression, TKI resistance, and relapse of disease. One of the approaches centers on modeling CML in appropriate* in vivo* mouse models in order to study the molecular pathogenesis of this disease and develop and improve therapeutical approaches. This review summarizes the recent advances, current challenges, and ongoing research in establishing mouse as well as a human xenograft* in vivo* model. Advantages and limitations of upcoming approaches such as iPSC technology and humanized xenograft mouse models for CML will be discussed as well (Figure 1) (Table 1).

## 2. Transgenic Mice Models

### 2.1. Conventional Models

The first transgenic model to assess the oncogenic potential of BCR-ABL was developed by introducing a synthetic BCR-ABL gene into the mouse germ line. A fusion of* bcr* and* v-abl* was expressed under the control of the immunoglobulin heavy-chain enhancer (E*μ*) or to the promoter/enhancer of the long terminal repeat (LTR) of the myeloproliferative sarcoma virus (MPSV) [27]. The enhancers of both constructs were capable of functioning in diverse hematopoietic cells including B/T lymphocytes as well as some myeloid cell lines, reasoning that they might be also functional in primitive HSCs from which these progenitors arise. The expression of the transgene during early development resulted in decreased offspring yield. Furthermore, only 3 out of 12 mice bearing E*μ*-driven BCR-ABL expression succumbed to pre-B and T lymphomas, while 1 out of 3 mice harboring MPSV LTR-driven BCR-ABL expression developed T lymphomas. No myeloid leukemias were developed with either construct. Another disadvantage was that BCR-v-ABL differed from BCR-ABL in that it lacked parts of the* bcr*- and* abl*-derived regions and it had several amino acid substitutions in the latter. Thus, the results obtained with the p210 BCR-v-ABL transgenic mice may not accurately reflect the biological properties of the original hybrid protein [27].

A more consistent outcome was achieved by using the delta metallothionein-1 promoter. This promoter is constitutively active in all tissues and was used to drive expression of the p190 BCR-ABL gene. Ten out of 60 p190/deltaMT transgenic mice were obtained, out of which 8 succumbed to death due to myeloid (2/8 mice) and lymphoid (6/8 mice) leukemia between 10 and 58 days of birth [28]. In follow-up studies with a bigger cohort of mice transplantable ALL/lymphoma was shown with same construct [28, 29]. Furthermore, in animals expressing the p210 BCR-ABL construct under control of the MT promoter T cell leukemias developed with no signs of B-ALL as was seen in p190 BCR-ABL mice [30].

In another study, the same group used the tec promoter to drive p210 BCR-ABL expression in transgenic mice. The tec gene encodes for a cytoplasmic kinase that is preferentially expressed in hematopoietic precursor cells. Using this transgenic model, 5 founders were generated, out of which 2 developed ALL shortly after birth, while transgenic progeny exhibited MPDs resembling human CML after much longer latency of 4–8 moths [31]. These results indicated that tec-driven p210 BCR-ABL transgenic mice could exhibit fundamental features of CML malignancy.

### 2.2. Conditional Models

Huettner and colleagues took a new approach by establishing a conditional transgenic mouse model for CML [32]. Transgenic mice with p210 BCR-ABL under control of a tetracycline response element (TRE) were generated and offspring of 4 such transresponder transgenic mice were mated with transactivator mice (tTA under control of MMTV-LTR) under continuous administration of tetracycline in the drinking water, starting 5 days prior to mating. Double transgenic mice were obtained with the estimated Mendelian frequency in all 4 lines. Upon tetracycline withdrawal, BCR-ABL1 was expressed and 100% incidence of lethal B-ALL was observed without any signs of myeloid or T cell malignancy. This was probably due to B-cell type specific promoter, the MMTV-LTR directing expression of tTA to B220^+^ cells. However, this study was the first of its kind to show that blast cell counts in advanced-stage leukemic mice were reduced upon tetracycline administration to block BCR-ABL expression. These data indicated that BCR-ABL1 was important for both the induction and the maintenance of the disease in these mice. Nevertheless, all reverted mice from one of the founder lines did succumb to BCR-ABL1-independent B-ALL in 2–4 weeks probably due to acquisition of secondary mutation(s) during disease progression [32].

Later studies from the same group further modified this tet-off inducible transgenic model by placing tTA expression under control of the 3′ enhancer of the murine stem cell leukemia (*scl*) gene [33]. SCL is a critical regulator of hematopoiesis and is normally expressed in erythroid and megakaryocytic cells, mast cells, and multipotent stem/progenitor cells. In these mice, induction of BCR-ABL resulted in neutrophilia and leukocytosis, bone marrow hyperplasia, and extramedullary myeloid infiltration, thereby recapitulating many features of CML [33]. Furthermore, 31% of animals with myeloproliferative disease progressed to B-ALL [33]. Also, a reversion of the myeloid and lymphoid phenotypes was shown upon loss of BCR-ABL expression, although further experiments were required to assess whether loss of BCR-ABL can be tolerated during advanced stages of disease.

### 2.3. Homologous Recombination Approach

Homologous recombination was used as an alternative approach to create p190 BCR-ABL transgenic mice by inserting the BCR-ABL cDNA into exon1 of the mouse* bcr* locus. The construct was electroporated into ES E14 cells, thereby generating ES cells that contained 1 intact bcr allele and another rearranged allele due to the BCR-ABL fusion. When these correctly modified ES cells were injected into recipient C57BL/6 blastocytes, all chimeric mice developed B-ALL. Similar leukemia phenotypes were observed in 37 out of 40 chimeric mice that were obtained in the absence of the endogenous bcr allele [35]. This strategy was very useful for studying the leukemogenic potential of p190 BCR-ABL expressed under normal transcriptional control elements.

In summary, transgenic models were first of their kind to validate the oncogenic potential of BCR-ABL and to support the expression of BCR-ABL as the prime initiating event for CML induction by p190 BCR-ABL [29]. Furthermore, conditional models largely contributed to understanding the course of human CML as well as studying CML leukemic stem cells. It was using this inducible scl/p210 BCR-ABL transgenic murine model that the studies from group of Sengupta et al. confirmed the role of BMI1 in collaboration with BCR-ABL to transform chronic phase lymphoid progenitors to induce serially transplantable B-ALL [34]. The use of transgenic (conditional) models can thus play a major role in understanding secondary collaborating events in the progression of CML. However, generation of new founder transgenic mice every time as well as BCR-ABL toxicity and silencing for establishing CML transgenic models remains a cumbersome and challenging effort.

## 3. Transduction/Transplantation Models

A murine bone marrow transduction approach was set up by Daley and colleagues with a retrovirus encoding p210 BCR-ABL in order to model CML [5]. Transduced cells were transplanted into 30 lethally irradiated syngeneic recipients. 13 out of 30 (43%) transplanted mice developed 3 distinct hematological malignancies within 5 months after reconstitution: a CML-like myeloproliferative syndrome with a mean latency of approximately 9 weeks, ALL with a mean latency of approximately 14 weeks, and a tumor containing macrophage cell types with a mean latency of approximately 16.5 weeks [5]. Bone marrow from one of the affected mice was transplanted into lethally irradiated secondary recipients that also developed a CML-like syndrome [5].

Kelliher and colleagues also transduced bone marrow cells from 5-FU treated mice with either* v-abl* or p210 BCR-ABL retroviruses and transplanted these into lethally irradiated mice. Half of the mice developed a myeloproliferative disease resembling chronic phase CML, while the remaining animals developed pre-B-cell lymphomas [36]. All 19 mice that received Abelson murine leukemia virus (Ab-MLV) infected cells succumbed 4–10 weeks later due to myelomonocytic leukemia and pre-B-cell lymphoma, while 11 out of 12 mice that received BCR-ABL transduced cells died 9–12 weeks after reconstitution due to myelomonocytic leukemia, granulocytic leukemia, and pre-B-cell lymphoma [36]. A great asset of this transduction model was that clonality could be assessed by making use of the unique integration sites as clonal markers.

Follow-up studies by several groups in which transduction efficiencies were further improved led to retroviral transduction/transplantation models that resulted in a rapid induction of CML-like myeloproliferative disease (MPD) in 100% of the cases, possibly due to efficient BCR-ABL expression in appropriate target cells. This disease was characterized by increased granulocytosis, splenomegaly, dissemination to other organs (lung), and efficient secondary transplantations [37–40]. However, variability in viral titers still affected the reproducibility of such models. Also, the use of strong promoters to drive BCR-ABL expression hindered modeling of leukemia with longer latency.

The transduction/transplantation models prove to be more efficient as compared to transgenic mice in terms of quick and simple approach which facilitate easy determination of clonality of leukemias and secondary transplantations as well as studying collaborating events synergistic for BCR-ABL induced disease. Studies from Bousquet et al. showed that miR-125b overexpression independently induced lymphoid or myeloid leukemia as well as acting as a secondary event by accelerating the development of p210 BCR-ABL induced leukemia [41]. Studies from Chang et al. have highlighted the role of Vav3 as critical GEF in p190 BCR-ABL mediated activation of RAC GTPase and downregulation of proapoptotic signals required for leukemogenesis [42]. Although current murine transduction/transplantation models fail to mimic the longer latency of human CML and transplantable myeloid secondaries, it is of utmost importance to validate results of such murine models by using appropriate xenograft models.

## 4. Xenotransplant Models

One of the earliest attempts to model human CML in immunodeficient mouse models dates back to the late '70s, where the BCR-ABL^+^ cell line K562 was injected into nude mice which grew as solid vascularized tumors containing cells like those seen in the patient and in the cultures [43]. Similar observations with the K562 cell line were later reported by Caretto and colleagues [44]. Sawyers and colleagues observed that cell lines and BM samples from patients with myeloid blast crisis CML could be transplanted into SCID mice by injecting the cells into a localized area, either by intraperitoneal (i.p.) injection or within the renal capsule [45]. Efficient engraftment and dissemination of leukemia was observed in BM and PB. Interestingly, differential growth in the spleen was seen in these myeloid blast cells as compared to lymphoid cells. Engraftment of chronic phase CML patient samples was infrequent and only limited myeloid growth was observed [45]. Sirard et al. also provided evidence of engraftment of both normal and leukemic hematopoietic cells from CML patients in sublethally irradiated SCID mice without exogenous treatment of cytokines [46]. However, high cell numbers for transplantation and infrequent and limited lymphoid as well as myeloid outgrowth were still a drawback in these SCID mice, perhaps due to residual innate immunity of these SCID mice.

Over the years, with the development of better immunodeficient models such as the severe combined immune deficiency (SCID) mouse model, transplantation of human normal and leukemic cells improved significantly. In particular, improvement of engraftment of lymphoid cells was achieved. However, engraftment of myeloid (malignant) cells remained challenging and many researchers focused their efforts on developing a system that would permit efficient interactions between human cells and their microenvironment. A first model that attempted to recreate a human environment to allow long-term multilineage human hematopoietic activity was reported by McCune and colleagues by coimplantation of small fragments of human fetal thymus and fetal liver into immunodeficient SCID mice, although this model was not used to study myeloid transformation [55]. With the development of NOD-SCID mice with functionally defective lymphocytes and NK cells several groups successfully showed engraftment of mononuclear and CD34^+^ cells from PB of CML patients using lower cell numbers than those reported for SCID mouse models of CML [47–49]. One of the challenges in developing* in vivo* models for human CML that remained was that normal stem cells frequently outcompeted chronic phase CML stem cells. By identifying patients in which the* in vitro* long-term culture-initiating cells (LTC-ICs) were predominantly leukemic and by injecting those cells into NOD-SCID or NOD-SCID *β*2 m^−/−^ mice more consistent engraftment was seen for 5 months after transplantation [8]. At late time points mice succumbed to endogenously derived thymomas, but no indication of progressive disease was evident, indicating that this model only represented the chronic phase of the disease [8].

The group of Connie Eaves presented a preleukemic* in vivo* model for CML by transducing primary human cord blood CD34^+^ cells with MSCV driven p210 BCR-ABL followed by transplantation into NOD-SCID and NOD-SCID *β*2^−/−^ mice. Increased engraftment was observed with a reduction in B-lineage output and enhanced production of erythroid and megakaryocytic cells [50]. However, only 4 out of 28 mice displayed elevated WBC counts and/or gross splenomegaly with a latency of 5-6 months. Although progression to blast crisis CML was not observed, these data clearly indicated that expression of BCR-ABL resulted in deregulated growth and differentiation which in some cases progressed to early stage disease.

Since BCR-ABL by itself appeared to be insufficient to induce leukemia in xenograft models, it has been proposed that additional pathways might be necessary to cooperate with BCR-ABL in the transition from CP-CML into BC-CML, such as the Wnt and Hedgehog pathways [16, 56, 57]. Alternatively, it has been observed that expression of the polycomb gene BMI1, which is implicated in normal and leukemic stem cell proliferation [58, 59], is significantly higher in patients with advanced disease than in patients in CP [60]. Therefore, we evaluated whether BMI1 could act as a collaborating factor together with BCR-ABL. We confirmed that coexpression of BMI1 and BCR-ABL in human CB CD34^+^ cells is sufficient to induce transplantable leukemia in NOD-SCID mice [51]. The disease progressed to lymphoid blast crisis phase and serially transplantable B-ALL could be established [51].

Although the development of xenograft mouse models has allowed the ability to work with primary patient samples* in vivo*, the pathogenesis of CP-CML progressing into a BC-CML was still very difficult to model. And while in human retroviral transduction models a serially transplantable B-ALL could be established, the development of myeloid malignancies in xenograft models remained rather challenging. Various phenomena could underlie these observations including residual host immunity in the xenograft models that were used or possibly even more importantly the lack of a proper human microenvironment for sustaining human myeloid leukemias.

## 5. Humanized Mice Models

The most immune deficient mouse model till date is the IL2R*γ* ^−/−^ mouse (NSG), which features the NOD/ShiLtJ background, the severe combined immune deficiency mutation (scid), and loss of the IL-2 receptor gamma and completely lacks T, B, and NK cells. After their development, NSG xenograft transplantation models soon emerged as the gold standard for accessing* in vivo* repopulation of HSCs as well as LSCs. However, in 2011, the group of Dominique Bonnet reported the failure of engraftment of 60–70% primary AML cells [61]. Importantly, these NSG mice remained rather biased towards lymphopoiesis, and normal or malignant myelopoiesis remained difficult to achieve. Despite the fact that these xenograft models are immunodeficient and would tolerate a human graft, the absence of a human microenvironment might still prevent appropriate growth of human cells. Indeed, various growth factors are species-specific, and, for instance, murine IL-3 or GM-CSF fail to activate human receptors, which might, at least in part, explain the lymphoid bias. The group of Donna Hogge and Connie Eaves established NOD-SCID mice transgenic for human SCF, IL-3, and GM-CSF [62, 63]. A number of AML samples that failed to engraft in the regular NOD-SCID mice could now engraft in the human cytokine NOD-SCID mice. The group of Jim Mulloy then crossed these mice into the NSG background and also reported better engraftment of AML samples in NSG transgenic for hSCF, hIL-3, and hGM-CSF [64].

Although these mouse xenograft models clearly hold promise, there are some potential pitfalls also. Since these are transgenic models and the human alleles are not knocked into their endogenous loci, expression levels might differ from the physiological levels, which might have important consequences for the normal self-renewal and differentiation programs. Possibly, this is tackled in the MYSTRG mice generated by the Flavell lab, in which 4 human cytokines are knocked into their respective mouse loci [65]. However, an appropriate niche obviously consists of more factors than just these cytokines. Direct interactions with the niche not only are important for normal HSCs but also are critical for LSCs [66]. For instance, N-cadherin mediates critical interactions between CML LSCs and their niche [67]. In fact, there is a continuous crosstalk between LSCs and their niche and in particular in the case of CML it has been documented that LSCs gradually change their niche so that it favors leukemogenesis [68, 69]. Thus, an ultimate model in which human hematopoiesis and leukemogenesis might be studied is a model in which the niche is of human (malignant) origin as well. Richard Groen has described a humanized model using NSG mice in which ceramic scaffolds seeded with human mesenchymal stromal cells were implanted to generate a human bone marrow- (huBM-) like niche [70, 71]. We have extensively studied our retroviral CB CD34^+^ BCR-ABL model within these humanized niche NSG mice as well, and our data indicate that BCR-ABL overexpression alone was sufficient to induce both AML and ALL, which could be serially transplanted [72]. By comparing transcriptomes of leukemias derived from murine niches versus leukemias from huBM-like scaffold- (huBMsc-) based niches, we observed striking differences in expression of genes associated with hypoxia, mitochondria, and metabolism. Efficient engraftment of blast-crisis CML patient cells was also observed, whereby the immature blast-like phenotype was maintained in the human scaffold niche but to a much lesser extent in murine niches. Thus, we have established human niche models in which the myeloid and lymphoid features of BCR-ABL^+^ leukemias can be studied in detail [72]. Future studies will be aimed at determining whether chronic phase CML patient samples can also engraft in this model and whether the progression from chronic phase to blast crisis CML can be modeled as well.

## 6. Conclusion

CML is one of the most studied and best understood hematological malignancies until now and has captivated the interest of several cancer stem cell enthusiasts to push the limit of drug discovery by further understanding the molecular mechanisms underlining this disease. Over the past 35 years, CML models have been developed and continuously improved, which have greatly aided in our understanding of the disease. With the establishment of a humanized niche model, it will be possible to further study and understand the crosstalk between leukemic cells and their environment in relation to the pathogenesis of CML. This should bring us a step closer to a complete understanding of the mechanisms by which BCR-ABL exerts its transformation potential, intrinsically within hematopoietic cells as well as extrinsically by modifying its niche.

## 7. Future Prospects

Apart from regenerative medicine, iPSC technology is also providing an excellent platform to model and study the pathophysiology of diseases such as myeloproliferative neoplasms including CML and PV. Carette et al. successfully reprogrammed the KBM7 BC-CML cell line into CML iPSCs [52]. Hu et al. reprogrammed MNCs from BM of CP-CML patient using nonintegrating episomal vectors [53]. Although the DNA methylation pattern of CML-iPSCs was different from that of original CML samples, it was iPSC-like; it was also very similar to normal iPSCs and human ES cells in terms of gene expression profile. However, the disease never progressed to BC phase probably due to lack of additional mutations. Later on, the group of Kurokawa generated CML iPSCs from imatinib sensitive CML patient samples [54]. Intriguingly, the CML-iPSCs were insensitive to imatinib, while CML-iPSC-derived hematopoietic cells recovered the sensitivity to imatinib with the exception of CD34^+^38^−^90^+^45^+^ immature cells which remained resistant, possibly in line with what is observed in patients. A challenge that remains is that reprogramming of CML iPSCs results in epigenetic alterations different from what was observed in the original patient samples and thereby also the characteristics of the iPSC CML cells. Despite this, iPSC provides an exciting novel technology with which various aspects of CML can be studied and novel specific targeted therapies can be developed.

## Figures and Tables

**(a):** 

Mouse models	Method	Phenotype of leukemia	Features	References
Conventional transgenic	Synthetic BCR-v-ABL driven by E*μ* or promoter/enhancer of MPSV LTR	Pre-B and T lymphomas (3/12 with E*μ*Vh vector), T lymphomas (1/3 with MPSV LTR vector)	BCR-v-ABL possesses oncogenic capability *in vivo*. Synthetic BCR-v-ABL was not similar to original hybrid protein. Increased embryonic lethality	[27]

Conventional transgenic	p190 BCR-ABL gene driven by delta metallothionein-1 promoter	Myeloid leukemia (2/8 mice) and lymphoid leukemia (6/8 mice) between 10 and 58 days of birth	Follow-up studies with bigger cohort developed transplantable ALL/lymphoma	[28, 29]

Conventional transgenic	p210 BCR-ABL gene driven by delta metallothionein-1 promoter	T cell leukemia	The tumorigenicity of p210 BCR-ABL chimeric gene products is specific for the hematopoietic cells	[30]

Conventional transgenic	p210 BCR-ABL gene driven by the mouse tec promoter	ALL (2/5 founder mice developed), MPDs in transgenic progeny with 4–8 months of latency	Transgenic progeny of one founder mice exhibited MPD with fundamental features of CML. Secondary mice showed excessive proliferation of myeloid and megakaryocytic cells; however, they succumbed to progressing anemia	[31]

Conditional transgenic	Tet-off system: tTA driven by the MMTV-LTR promoter	Lethal B-ALL developed in 100% of mice within 3–11 weeks on withdrawal of tetracycline due to p210 BCR-ABL1 expression	MMTV-LTR promoter directed expression of tTA to B220^+^ BM cells. Abolition of BCR-ABL1 expression led to apoptosis of leukemic cells and hence reversal of B-ALL phenotype. Reverted mice from one founder did succumb to ALL without BCR-ABL expression, possibly due to the acquisition of additional mutations	[32]

Conditional transgenic	Tet-off system: tTA driven by the SCL promoter	Neutrophilia, leukocytosis, and dissemination of myeloid cells into spleen, liver, and lymph nodes within 29–122 days upon tetracycline withdrawal due to p210 BCR-ABL1 expression. 31% of mice progressed to B-ALL	SCLtTA/BCR-ABL expression model recapitulates many features of human CP-CML	[33]

Conditional transgenic	Overexpression of BMI1 by ubiquitin C promoter in a lentiral EGFP vector in BCR-ABL expressing CP-CML stem cells and progenitor using Scl/p210 BCR-ABL binary mouse model	Development of serially transplantable B-ALL with accumulation of BMI1/BCR-ABL^+^ B-cells after 16 weeks of transplantation	BMI1 synergizes with BCR-ABL to transform chronic-phase SCL/p210 B-lymphoid progenitors but not HSCs or multipotent progenitors (MPPs) and imparts a proliferative advantage to induce serially transplantable B-ALL *in vivo*	[34]

Transgenic by homologous recombination	E14 ES cells to create in-frame fusion of p190 BCR-ABL with exon 1 of murine *bcr* in the presence or absence of the endogenous nonrearranged *bcr* allele	*bcr*-*ABL*/*bcr* ^+^ chimeric mice and 37/40 *bcr*-*ABL*/*bcr* ^−^ developed B-ALL	*bcr*-*ABL* fusion gene does not require endogenous *bcr* allele to develop leukemia	[35]

Transduction/transplantation	Mu BM transduced with retroviral construct of p210 BCR-ABL expressed under control of MPSV myeloid cell-specific promoter	13/30 developed 3 distinct malignancies: CML, ALL, and macrophage-like tumor	Retrovirus mediated expression of p210 BCR-ABL demonstrates murine model system for CML	[5]

Transduction/transplantation	Mu BM of 5-fluorouracil (5-FU) treated mice transduced with v-abl or p210 BCR-ABL retrovirus	>90% mice that received v-abl or BCR-ABL transduced cells died due to myelomonocytic leukemia, granulocytic leukemia, and pre-B-cell lymphoma	Both BCR-ABL and activated v-abl can induce similar malignancies. Integration site analyses allowed evaluation of clonality	[36]

Transduction/transplantation	Mu BM transduced with retroviral construct of p210 BCR-ABL under control of MSCV LTR promoter in the presence of SCF	All recipients came down with disease and displayed markedly elevated WBC counts with granulocyte predominance	Induction of murine CML in 100% of recipients with 4–6 weeks of latency. All secondaries succumbed to lymphoid neoplasms.	[37]

Transduction/transplantation	5-FU treated Mu BM cells transduced with retroviral construct of p210 BCR-ABL expressed under control of MSCV promoter	Elevated WBC counts, majority of which were granulocytes but also included myeloblasts and basophils	Induction of transplantable myeloproliferative disease resembling CML. Leukemic cells expressed excess IL3 and GM-CSF	[38, 39]

Transduction/transplantation	5-FU treated/untreated Mu BM cells transduced with retroviral construct of p210 BCR-ABL, p190 BCR-ABL, and p230 BCR-ABL expressed under control of MSCV promoter	CML-like syndrome when 5-FU treated donor cells were used. Mixture of CML-like disease, B-ALL, and macrophage tumors when non-5-FU treated donor cells were used	All 3 forms of BCR-ABL induce identical CML-like syndrome in mice but p190 BCR-ABL had increased potency for induction of B-ALL	[40]

Transduction/transplantation	5-FU treated bone marrow cells with a retrovirus encoding p210 BCR-ABL together with the XZ-miR-125b overexpressing miR-125b or the control vector XZ were transplanted into lethally irradiated BALB/c recipient mice	50% of mice died of B-ALL, 42% with MPN, and 8% of mixed (myeloid and lymphoid) leukemia when transplanted with miR-125b plus BCR-ABL-infected cells with median survival of 21 days as compared to 35 days in BCR-ABL transduced control group	miR-125b accelerates the oncogenicity of BCR-ABL in transplanted mouse model	[41]

Transduction/transplantation	B-ALL LDBM cells from specific gene-deleted murine models or WT mice and UCB CD34^+^ were transduced with bicistronic vectors expressing EGFP and p190-BCR-ABL (MSCV-p190-BCRABL) or only EGFP (MIEG3) and then cultured with IL-7 and SCF	>90% of recipient mice developed B-ALL in approximately 37 days characterized by B220^dim+^, CD19^+^, and CD43^+/dim^ B-cell progenitor population. Infiltration in other organs was also evident	Vav3 plays a crucial role in p190-BCR-ABL-mediated leukemogenesis, proliferation, and survival especially for the B-cell progenitor	[42]

**(b):** 

Human models	Method	Phenotype of leukemia	Features	References
Xenograft nude mice	Nude mice injected with K562	K562 grew as solid vascularized tumors	Tumor cells were triphoid and retined human chromosome markers	[43]

Xenograft nude mice	SIA-nu/nu mice were injected with leukemic cell lines and primary patient sample	K562 formed solid tumor at challenged site without metastatic spread with mean latency of 10 days	6/8 leukemic cell lines and 5/18 primary neoplastic tumors induced serially transplantable solid soft mass	[44]

Xenograft SCID mice	BM samples of CP-CML and BC-CML as well as cell lines; K562 and EM-2 were transplanted into CB-17 *scid*/*scid* mice	All mice injected with K562 as well as EM-2 or primary CP-CML and BC-CML samples by IV or IP engraft to give myeloblasts in BM, blood, and tumors in peritoneum	After initial growth in kidney capsule, myeloblasts were detected at varying levels in PB and BM. Human myeloid and lymphoid leukemia cell lines showed distinct growth patterns. Differences were also observed in engraftment of CP versus BC-CML primary patient samples	[45]

Xenograft SCID mice	BM or PB samples obtained from CP-CML and BC-CML patients were injected by IV into sublethally irradiated [400 cGy] SCID mice. Exogenous cytokines PIXY321 or c-kit ligand was injected IP	CP-CML and BC-CML patient sample showed 1–>10% engraftment with 30–60 days of latency in presence or absence of exogenous human cytokines	Multilineage engraftment and CD34^+^ cells were maintained for more than 60 days after transplantation. First evidence that both normal and leukemic CP-CML cells can engraft in SCID mice	[46]

Xenograft NOD/SCID	MNCs from apheresis material from CML patient were IV transplanted into sublethally irradiated [300 cGy] NOD/SCID mice. Preselected CD34^+^ and CD34^−^ cells were also used for BM engrafted studies	≥1–84% multilineage engraftment observed in BM in 76% mice and only 66% of mice showed 16% predominantly T cell splenic engraftment. CML-like disease in BM and spleen. 39% ± 5% leukemic engraftment in 25 mice having ≥9% BM engraftment was higher as compared to BCR-ABL engraftment in spleen	Higher engraftment in NOD/SCID mice using low cell dose compared to SCID mice	[47]

Xenograft NOD/SCID	MNCs or CD34^+^ enriched cells from BM or PB of 11 CP-CML patient were IV transplanted into sublethally irradiated [400 cGy] NOD/SCID mice	25% of NOD/SCID recipients had 40–80% human cells, whereas only 3% SCID mice contained similar levels. Further, engrafted human cells had high proportion of leukemic cells along with CD34^+^ cells	NOD/SCID mice allow greater engraftment and amplification of both normal and leukemic cells as compared to SCID mouse model	[48]

Xenograft NOD/SCID	BV173 and PB MN cells from CP, AP, and BC CML patient samples were injected	Kinetics and extent of engraftment BP > AP > CP, although according to growth rate BP > AP ≥ CP	Kinetics of BM repopulation are different for CP, AP, and BC phase of CML	[49]

Xenograft NOD/SCID	9 CP-CML patient samples with predominant LTC-IC were transplanted into sublethally irradiated [350 cGy] NOD/SCID and NOD/SCID *β*2m^−/−^ mice	Consistent and durable engraftment was observed with reduced output of B cells and enhanced myelopoiesis with excessive production of erythroid, megakaryocytes, and BCR-ABL CD34^+^ expressing IL-3 and G-CSF transcripts	No progressive disease phenotype was observed marking CP-CML phase of the disease	[8]

Xenograft NOD/SCID	CB CD34^+^ cells were transduced with MSCV based retroviral constructs for BCR-ABL and transplanted 0.2 to 0.3 million cells into each sublethally irradiated [350 cGy] NOD/SCID and NOD/SCID *β*2m^−/−^ mice	BCR-ABL transduced cells produced increased ratio of myeloid to B-lymphoid cells with increase in erythroid and megakaryocytic cells. 4/28 mice developed an increased WBC count and/or splenomegaly after 5-6 months of latency	First ever model to describe the *de novo* generation of preleukemic cells by forced expression of BCR-ABL in human CD34^+^ CB cells. Primary CD34^+^ CB cells showed rapid and persistent deregulation and erythroid and megakaryocytic biased differentiation *in vivo* with occasional progression to an early stage of CML	[50]

Xenograft NOD/SCID	CB CD34^+^ cells were transduced with MSCV based retroviral constructs for BMI1 and BCR-ABL and transplanted only 0.46 to 0.38 million cells into each sublethally irradiated [3 Gy] NOD/SCID mice	4/8 mice succumbed to [CD34^+^ CD19^+^] B-ALL in 16–22 weeks on transplantation of CD34^+^ cells cotransduced with BMI/BCR-ABL and all secondaries came down with similar phenotypes within 8–12 weeks	Coexpression of BMI1 and BCR-ABL in CB CD34^+^ cells is sufficient to induce transplantable B-ALL in NOD/SCID mice	[51]

iPSC	KBM7 cells were reprogrammed by retroviral transduction of *OCT4*, *SOX2*, *c-MYC*, and *KLF-4*	Teratoma formation and imatinib resistance were observed	The process of reprogramming KBM7 cell lines readily abolished BCR-ABL dependency which was restored by differentiation into hematopoietic lineage	[52]

iPSC	MNCs from BM of CP-CML patient sample were cultured with human SCF, IL-3, IL-6, and Flt3L for 2 days and transfected with episomal vectors by nucleofection	CP-CML iPSC lines generated exhibited features of pluripotent stem cells, exhibited complex karyotype, and differentiated into hematopoietic lineages	Transgene free CML iPSC lines can be obtained	[53]

iPSC	Regeneration of CML iPSCs from CD34^+^ BM MNCs of CP-CML patient sample by retroviral vectors	DNA methylation pattern and gene expression profile of CML-iPSCs were different from those of original CML sample but were similar to normal iPSCs and human ES cells	Recapitulation of CP CML was shown in terms of that fraction of phenotypically immature cells which showed imatinib resistance although more differentiated cells recovered the sensitivity to imatinib	[54]
